# Supplementary figures and images for: A lower psoas muscle volume was associated with a higher rate of recurrence in male clear cell renal cell carcinoma
Source: PLoS One. 2020 Jan 2;15(1):e0226581. doi: 10.1371/journal.pone.0226581 (PMC6939903; doi:10.1371/journal.pone.0226581)

## Slide 1
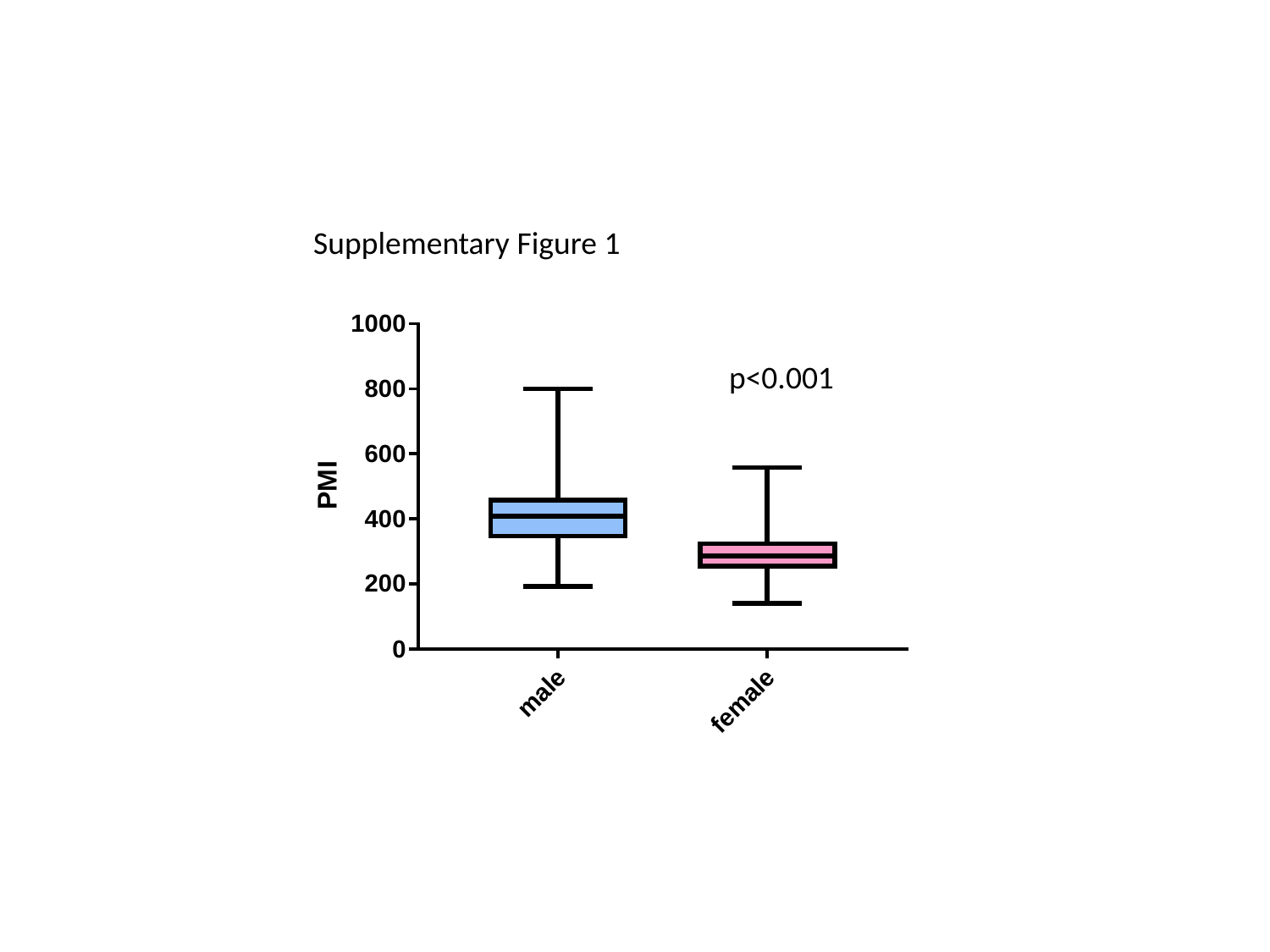

Supplementary Figure 1
p<0.001

Supplement: S1 Fig — (PPTX) [file pone.0226581.s001.pptx]
