# Supplementary material for: A lower psoas muscle volume was associated with a higher rate of recurrence in male clear cell renal cell carcinoma
Source: PLoS One. 2020 Jan 2;15(1):e0226581. doi: 10.1371/journal.pone.0226581 (PMC6939903; doi:10.1371/journal.pone.0226581)
Supplement: S1 Table — (DOCX) [file pone.0226581.s002.docx]

| Supplementary Table 1 | | |
| --- | --- | --- |
|  |  | median (mean ± SD), number (%) |
| Male |  | 11 (100.0%) |
| Age (yrs.) |  | 65 (63.0 ± 13.8) |
| Side | Rt | 6 (54.5%) |
|  | Lt | 5 (45.5%) |
| Tumor size (cm) | | 4.1 (4.9 ±2.9) |
| Body height (cm) | | 166.0 (167.7 ±6.35) |
| PMI |  | 455.6 (442.3 ±118.4) |
| PMV |  | 153.4 (169.7 ±59.5) |
| PMI: psoas muscle index, PMV: psoas muscle volume | | |
