# Supplementary material for: A lower psoas muscle volume was associated with a higher rate of recurrence in male clear cell renal cell carcinoma
Source: PLoS One. 2020 Jan 2;15(1):e0226581. doi: 10.1371/journal.pone.0226581 (PMC6939903; doi:10.1371/journal.pone.0226581)
Supplement: S2 Table — (DOCX) [file pone.0226581.s003.docx]

| Supplementary Table 2 | | | | |
| --- | --- | --- | --- | --- |
|  |  | Recurrence (n=49) | No recurrence (n=267) |  |
|  |  | n (%) | n (%) | p |
| Age | ≤60yrs. | 13 (26.5%) | 112 (41.9%) |  |
|  | >60yrs. | 36 (73.5%) | 155 (58.1%) | 0.042 |
| Site | Right | 25 (51.0%) | 143 (53.6%) |  |
|  | Left | 24 (49.0%) | 124 (46.4%) | 0.744 |
| PS | 0 | 40 (88.9%) | 252 (94.7%) |  |
|  | ≥1 | 5 (11.1%) | 14 (5.3%) | 0.130 |
| Stage | 1 & 2 | 23 (46.9%) | 232 (86.9%) |  |
|  | 3 | 26 (53.1%) | 35 (13.1%) | <0.001 |
| Size | ≤4cm | 11 (22.4%) | 176 (65.9%) |  |
|  | >4cm | 38 (77.6%) | 91 (34.1%) | <0.001 |
| Grade | 1 & 2 | 24 (49.0%) | 212 (79.4%) |  |
|  | 3 & 4 | 25 (51.0%) | 55 (20.6%) | <0.001 |
| PMI | High | 18 (36.7%) | 140 (52.4%) |  |
|  | Low | 31 (63.3%) | 127 (47.6%) | 0.043 |
|  |  |  |  |  |
