# Supplementary material for: A lower psoas muscle volume was associated with a higher rate of recurrence in male clear cell renal cell carcinoma
Source: PLoS One. 2020 Jan 2;15(1):e0226581. doi: 10.1371/journal.pone.0226581 (PMC6939903; doi:10.1371/journal.pone.0226581)
Supplement: S3 Table — (DOCX) [file pone.0226581.s004.docx]

| Supplementary Table 3 | | | | | | | |
| --- | --- | --- | --- | --- | --- | --- | --- |
|  |  |  | Univariate | |  | Multivariate | |
|  |  | n | p | HR (95%CI) |  | p | HR (95%CI) |
| Age | ≤60yrs. | 125 |  |  |  |  |  |
|  | >60yrs. | 191 | 0.113 | 1.811 (0.869-3.773) |  |  |  |
| Site | Right | 168 |  |  |  |  |  |
|  | Left | 148 | 0.907 | 1.040 (0.535-2.023) |  |  |  |
| PS | 0 | 292 |  |  |  |  |  |
|  | ≥1 | 19 | 0.054 | 2.797 (0.980-7.979) |  |  |  |
| Stage | 1 & 2 | 255 |  |  |  |  |  |
|  | 3 | 61 | <0.001 | 6.775 (3.466-13.243) |  | 0.002 | 3.605 (1.616-8.043) |
| Size | ≤4cm | 216 |  |  |  |  |  |
|  | >4cm | 100 | <0.001 | 4.452 (2.289-8.660) |  | 0.104 | 2.051 (0.863-4.873) |
| Grade | 1, 2, & 3 | 302 |  |  |  |  |  |
|  | 4 | 14 | <0.001 | 17.105 (7.474-39.144) |  | <0.001 | 8.446 (3.465-20.586) |
| PMI | High | 158 |  |  |  |  |  |
|  | Low | 158 | 0.022 | 2.306 (1.129-4.708) |  | 0.02 | 2.338 (1.143-4.783) |
|  |  |  |  |  |  |  |  |
