# Supplementary material for: A lower psoas muscle volume was associated with a higher rate of recurrence in male clear cell renal cell carcinoma
Source: PLoS One. 2020 Jan 2;15(1):e0226581. doi: 10.1371/journal.pone.0226581 (PMC6939903; doi:10.1371/journal.pone.0226581)
Supplement: S1 Data — (PDF) [file pone.0226581.s005.pdf]

Age	side 1rt, 2lt	PMI	PMI_5yr	PS	1open,2partial	size
49	2	191.9170003		0	2	1.8
68	2	207.9952438		–	1	10
79	2	221.0484122		0	2	
73	2	223.3883728	218.8729766	0	1	2.5
67	1	224.201405		0	1	4.8
51	2	233.4197486		0	1	7
64	1	241.0926871	275.37911	0	2	
22	1	251.6756986	205.9199255	0	2	1.8
52	2	254.6096211	259.3582838	0	1	6.7
80	2	257.7999844	311.9924239	0	1	1.5
60	2	262.9559638		1	1	2
68	2	264.522449		0	1	10.5
72	1	264.6987209	295.2153949	0	1	2.7
75	1	267.9743276		0	1	
70	2	268.1067817	273.6429209	0	1	4.5
62	1	268.765866		0	1	2.4
60	1	270.7606857		0	2	2.7
76	2	272.9039219	260.6217652	0	1	
78	1	273.3359375		1	1	7.5
62	1	277.6426666	318.0234043	0	1	6
76	2	278.7901305		0	1	5.7
62	1	280.2714157	297.5824734	0	2	1.5
59	1	280.4753086	277.9506173	0	2	
67	1	281.9501801	346.7178543	0	1	5
79	1	284.166435	291.8459872	0	1	3.1
76	2	284.5879065		1	1	4.2
55	1	284.9151995	303.044211	0	1	2.6
85	2	285.2929553		1	1	6
75	2	286.1206127		0	2	1.6
72	1	289.4740484		1	1	3.8
64	2	291.2273243		0	2	2.3
75	1	291.584567		0	2	1.6
61	2	291.7802646	283.9869156	0	1	3
76	2	291.7894925		–	1	
56	1	294.87236		0	1	3.9
51	2	295.8468068		0	2	2
70	1	299.6697241		1	1	8
63	2	300.1744126		0	2	3
83	2	301.4109013		0	2	2.1
76	2	301.9601915	320.2579995	1	1	7.5
63	2	304.2260425	385.0145303	0	2	1.7
74	1	305.2479085		0	1	3.5
71	1	307.3631877	292.9997097	0	1	4.5
49	1	307.9230485	321.5865017	0	2	2.3
78	2	308.2653924		1	2	2.5
72	2	308.3767108		0	1	5
45	1	309.351502		0	1	3
63	1	309.9920185	260.5435833	0	1	10
51	2	310.013905		0	1	3.2
73	1	310.8276644	317.7508503	0	1	3.8
65	2	311.6725047		0	2	2
59	2	311.6977958		0	1	3
78	1	314.0005981		0	1	10.1
46	1	315.8889963		0	1	10.5
88	1	316.6016629		0	1	5.5
76	2	319.0738859		0	2	1.8
73	1	323.7394606		0	2	1.2
69	1	324.405116		0	1	4.9

83	1	324.7474414		0	2	1.8
67	2	327.3287365		0	2	
74	2	327.7844219		0	1	
69	1	328.2330009		0	1	
66	1	328.3421086	387.26907	0	1	6.8
72	1	328.4478835	259.1575925	0	1	5.6
65	2	328.6139203		0	1	3.5
59	2	329.4672667	355.526927	0	1	0.9
62	2	331.5200159	313.4462941	0	1	5
77	2	332.0073462	324.1726354	0	1	2.5
51	1	332.7766802		0	1	3
71	1	333.3398438		0	2	3.3
45	2	334.5839905	304.0924744	0	2	1.1
68	2	335.1617116		1	1	8.5
79	1	337.9506489		0	1	4.8
69	1	338.3075304		0	1	5.9
40	1	338.6492769	376.3946281	0	1	4
67	2	339.8494569		0	1	3.1
61	1	339.8725786		0	1	3.8
43	2	340.160582		0	1	3.4
65	1	340.466827	317.0745725	0	1	4.2
68	1	341.4816997		2	2	5.7
63	2	341.5417164		0	1	4.7
68	1	341.5684951	417.4938184	0	1	3.9
79	2	342.7750865	273.0934256	0	2	5.5
70	2	343.2380411		2	1	8.5
78	1	344.7266483		0	1	3.9
74	1	345.2723296		0	2	3.2
72	1	345.7458517		0	2	2.3
63	2	348.3993804		0	1	3.4
71	2	349.0597065	415.2217912	0	2	2.4
61	1	352.7309458		0	2	2
39	1	353.3307798	346.4330173	0	1	
58	1	354.5709343		0	1	
75	1	355.0364881		0	1	2.1
51	1	355.0754639	450.4347172	0	1	
72	1	355.1721987		0	1	
69	2	355.4669219		0	1	3.5
58	2	356.2130375	411.7611681	0	1	3.5
78	1	357.0811571	377.4022815	0	1	3.6
53	2	358.9832539		0	2	3
75	2	360.157897	373.2291777	0	2	5
70	1	360.667445	416.1710775	0	1	5
58	2	360.6745339		0	1	2.5
77	1	362.7416577		0	2	
74	1	363.4135714		0	1	3.8
75	2	365.3681782		0	1	4.4
77	2	366.5196348		0	1	4.5
61	1	366.9824931	328.1593001	0	2	
78	1	369.5593837		0	1	7
65	1	370.5862149	415.5203065	0	2	2
65	1	370.8086771	435.6407255	0	1	
78	1	371.3360247		0	1	6
62	2	372.0012044	411.4531973	0	1	3.3
73	1	375.6210678	367.4504116	0	2	
70	1	376.3131259		0	2	3.4
81	1	377.1496786	397.0688705	0	1	2.5
73	1	377.4005632		0	1	
60	2	377.9453507	361.6805171	0	2	2.8

73	2	378.8400015		0	1	5
80	2	379.0319307		0	1	2.5
68	2	379.0594499	429.4818873	0	2	
44	1	381.8810364		0	2	5.3
64	1	382.8997584	340.9859599	0	1	4.3
61	1	384.9531529	318.0807555	0	2	2.8
71	2	385.801431	466.4391127	0	1	6
46	1	385.9648575	362.7352455	0	1	6.2
76	2	386.0304322		0	1	7.5
61	1	386.2302272	443.6654795	0	1	7
40	1	386.2384575		−	1	
65	2	386.732457		0	1	9
78	2	387.3538043	293.0019052	0	1	3.3
65	2	387.5757664	368.7564251	0	2	3.5
78	2	388.3088775	353.398015	0	1	9
78	1	391.8776066		0	1	10
62	1	392.669725		0	1	2.2
74	2	392.8413415		0	1	2.9
65	2	393.112576	441.8086475	1	1	8
67	1	393.4054221	421.1143374	0	2	3
71	1	393.7883508	354.4062418	0	2	1.7
38	1	395.8740364	371.6113729	0	2	2.5
44	1	397.2638034	389.3387615	0	1	2.5
47	2	398.7128778		0	2	1.5
60	2	399.2125635	400.3262256	0	2	2.6
74	2	399.5660786		0	1	4
74	1	399.5794915	375.3288839	0	2	2.3
33	1	399.9298339	370.1927896	0	1	3.1
62	2	399.9665378		0	1	2.8
58	1	400.7834715	463.0063866	0	2	1.5
31	1	401.4174668		0	2	2.7
57	2	401.9487589	412.9264044	0	1	5
65	2	402.1083563		0	2	1.8
52	1	404.9773726	350.170378	0	2	1.5
73	1	405.3332831		0	2	2.4
60	1	406.1738289	407.5339087	0	2	1.8
75	2	407.0546061		0	1	10
41	2	408.5311595	378.3391561	0	1	2.1
77	2	408.8485424	376.357739	0	1	3.3
66	2	409.0833212	410.9595007	0	1	2.8
68	2	409.618822		0	1	10
49	2	409.6639635		0	2	
53	2	410.3450634	443.8254506	0	1	4.8
75	2	412.4870938	371.6354672	0	1	
53	2	412.8005844		0	1	4.6
66	1	412.8962351		0	1	6
41	1	413.6838073	410.9610904	−	1	
81	1	415.047666		0	1	5.5
38	1	415.2418247	436.6763047	0	1	1.6
65	2	415.573921		1	2	2.8
59	1	416.1814657		0	1	3
70	2	416.8354097		0	1	2.8
64	2	417.2293278	388.693486	1	1	7.5
52	2	417.3973785		0	1	4.3
75	1	417.8410465	399.832971	0	1	4.6
85	2	418.0472479		0	1	4.2
63	1	418.7197232	497.5778547	0	1	
71	2	419.1441106		0	1	
73	1	420.7311277		0	2	2

72	1	424.1616162	422.2038567	0	2	7.7
75	1	425.1218011	417.8498209	0	2	
77	2	425.873084	332.6186523	0	1	
59	2	427.9042386	485.2825746	0	1	4
54	2	428.1004796	409.9605071	0	2	2.3
49	1	428.349481	490.1868512	0	1	4.5
55	1	428.8762324		0	1	9.5
74	2	429.0980312		0	2	
58	1	429.7193878		0	2	2
52	2	429.8395523		0	1	4
45	2	429.9894222	430.7530895	0	2	1.6
37	2	430.203364	472.144305	0	1	
47	1	430.9906277	423.7535058	0	2	1.5
37	1	431.0269388	444.4440816	0	2	3.4
68	2	431.5310694		0	2	1.7
52	1	433.7760672		0	1	2.3
44	1	434.0344757		0	1	
62	1	436.2011306	402.4524497	0	2	1.8
63	1	436.6439657		0	2	1.9
61	2	437.29487		1	1	18
57	2	438.8185372	442.6409168	0	2	
46	2	438.9537478	449.405116	0	1	
66	1	439.3591756		0	1	2.5
77	2	439.6778746		0	1	
62	2	439.8723781		0	2	1.4
60	2	440.6715822	436.5986785	0	2	2.5
42	2	440.7777029	441.5408853	0	1	4
46	2	441.9930714	511.2012734	0	1	
40	1	441.9973855		0	2	1.5
77	2	442.0463942		0	1	5.3
74	1	442.5345327		0	1	2.3
75	1	442.9560042		0	2	3
41	2	443.3808958		0	2	3.5
52	2	443.5628474		0	2	
62	2	444.7237666		0	1	3.8
57	1	446.2523566		0	1	3.5
68	2	447.1565244		0	2	2
61	1	447.1867232		0	1	
70	2	447.636072		2	1	6.4
57	1	448.0110193	380.7162534	0	2	3.1
63	1	448.2857435	447.3671401	0	1	4
60	2	448.566614	426.5435806	0	1	
63	2	450.4359091	441.1925353	0	1	5
67	1	450.9480964	459.6445568	0	2	1.4
77	2	451.1648914		0	1	10
46	2	453.0276266	480.987847	0	2	1.2
50	2	453.8643445	383.5230004	0	1	2
63	1	454.338261		0	2	1
65	1	454.5022151		0	2	1.4
67	2	456.2229948	406.0123762	0	1	5.5
77	1	457.5972094		0	2	2.1
61	1	457.8423965	480.030343	0	1	4.6
69	1	458.1258488	449.2530557	0	1	6
64	1	458.6084039	474.030394	0	2	2
59	2	459.8059762	464.227285	0	2	2.3
66	2	460.1689624		0	1	6.5
58	1	461.0299734		0	1	
46	2	462.4065306	588.4636735	0	2	
56	1	463.7769235	469.6421505	0	1	7.8

58	2	464.3190835		0	1	1.7
74	1	465.0120465	396.6978458	0	1	
75	2	465.1729384		0	2	3.2
40	2	466.2846346	446.4607749	0	2	3.1
39	2	466.689554	534.0594863	0	1	
69	1	467.966427	315.5218488	0	1	4
35	2	469.2032045		0	1	1.8
56	1	470.1212479	513.24217	0	1	
42	1	472.5310032		0	2	
68	2	472.83729	470.3548108	2	1	3.2
60	2	474.0038805		0	1	3.8
47	1	474.1988913		0	1	
64	1	476.4363993		0	2	3.7
66	1	480.9536106	486.0798098	0	2	
68	1	481.1134827		0	2	1.1
73	1	481.1951647		0	1	6.5
56	2	482.6910225		0	2	
58	1	485.1454817	247.2048128	0	1	1.6
60	1	485.2638419	483.4513184	0	1	0.7
61	2	485.8061929	507.5090221	0	1	2.5
81	1	486.3723134		0	2	2.5
68	1	486.7448169		0	2	1.5
69	2	487.4625214		0	2	3
72	1	488.850279	456.884627	0	1	4.5
62	1	490.4751103	533.8273472	0	1	2.2
61	1	492.0621175		0	1	5.5
60	1	492.5688634		0	1	8.5
44	2	493.2465886		0	2	4.5
46	2	494.1617307	493.3249317	0	2	2.5
68	1	494.9444218		0	2	3
35	2	496.9057296		0	1	4
64	2	498.3561556		0	1	3.6
62	1	499.6806503		0	2	2.1
69	2	502.0448171	510.3172524	−	1	4
59	1	502.519388	371.3233561	0	1	2.9
64	2	505.1790634		0	1	4
72	1	507.9929187		0	1	3.5
60	2	508.1252189		0	1	4.7
73	1	508.1747738	588.3240699	3	1	9.5
43	2	508.3512876	537.1601518	0	1	
43	1	511.4415905		0	1	1.4
65	2	517.0160915		0	2	
34	2	517.6444297	597.1112124	0	2	3
36	1	518.2407747	511.201063	0	1	3.7
58	1	518.8792396		0	1	4.5
54	1	518.9016186	529.9851847	0	1	4.5
45	2	520.2192458		0	1	2.3
56	1	520.7199904	507.2758633	0	2	2.5
55	1	521.4105173		0	2	
60	1	527.1048048		0	2	3.5
43	1	527.9726563	625.3789063	0	1	
68	1	530.1220888	482.6271331	0	1	4.4
43	1	534.5003897	530.3850677	0	2	1.7
58	1	539.497994	500.7259954	0	1	
62	1	540.7013126	560.7533203	0	2	4.5
46	2	541.7716637	512.0133413	0	1	3.9
41	1	543.9230776		0	2	
64	1	548.2886905		0	1	4.2
49	2	550.0341009	497.9042288	0	1	5.7

65	2	559.7697901	608.387119	0	2	2
55	2	564.9767464		0	2	2.5
39	1	568.1543039		0	2	2.2
33	2	570.83832		0	1	5.3
66	1	574.6471452	556.8707893	0	1	8
54	2	577.7450526		0	1	5.7
68	2	579.1297721		0	1	4.1
63	1	581.2007343		0	1	1.7
46	1	584.7892098	466.8377155	0	2	2.1
51	2	589.7351385		0	2	1.8
48	1	591.1245675	555.7058824	0	1	3.5
80	1	593.6112616		0	1	
57	2	600.7855046	557.6133871	0	1	2.8
59	1	601.6280236	575.1205586	0	1	4.7
53	1	611.4335317	465.2281746	0	1	9.5
59	2	622.40955	545.4104683	0	1	4.1
50	1	637.8046875	611.2070313	0	2	1.4
47	1	664.7491438		0	2	1.5
67	1	667.2567417	629.4050131	0	2	5
65	1	675.9889807	590.2809917	1	1	3
52	2	735.7537682		0	1	11
39	1	800.7655223	793.0109569	0	1	3.7

grade	stage	rec	rec_free_survival
2	1	0	43.07257614
2	2	1	3.975424648
2	1	0	19.81141374
2	1	0	122.8767618
3	1	1	20.53421822
4	3	1	3.351184414
2	1	0	79.77133095
2	1	0	124.2566613
3	1	1	64.0996156
1	1	0	73.75891185
2	1	0	44.7153136
3	3	1	23.65541939
2	1	0	104.0509906
2	3	0	16.29595558
2	3	0	120.0841082
3	3	0	38.27578276
2	1	0	53.32325788
3	1	0	62.75257088
4	3	0	3.581167658
2	1	0	90.35056017
4	3	0	28.12366528
2	1	0	123.7309853
1	1	0	82.59683937
2	1	0	96.62581726
3	3	0	72.28044814
3	1	0	0.755659231
2	1	0	80.52699018
3	1	0	59.76278871
2	1	0	59.86135296
1	1	0	58.81000099
1	1	0	27.56513454
2	1	0	19.12146401
2	1	0	61.17554292
4	3	1	4.172553143
2	1	0	9.987843743
2	1	0	6.143838092
4	3	1	5.42103361
2	1	0	29.60212899
2	1	0	19.12146401
2	2	0	91.1062194
2	1	0	169.0048296
2	1	0	9.33074876
2	1	0	62.55544239
1	1	0	65.70949831
1	1	0	20.36994448
3	3	0	32.13194467
1	1	1	49.15070473
1	3	0	84.96238131
2	1	0	4.599664882
1	1	0	112.3632421
2	1	0	6.406676085
2	1	1	6.965206821
3	2	1	6.373821336
3	2	0	59.03998423
1	1	0	62.1611854
2	1	0	8.673653777
2	1	0	20.43565397
2	1	1	42.74402865

2	3	0	0.78851398
2	1	0	54.4731741
2	1	0	44.5838946
3	1	0	12.22196669
3	3	0	122.3839406
1	1	0	102.9010744
2	1	1	26.61234681
2	1	0	138.0228012
2	1	0	69.58635871
1	1	0	74.41600683
2	1	0	30.78489996
2	1	0	43.49968788
2	1	0	70.07917995
3	3	0	2.266977692
3	3	1	7.458028058
2	1	0	3.614022407
2	1	0	113.6445773
2	1	0	0.689949732
2	1	1	37.75010678
2	3	0	25.03531886
3	3	0	60.68272169
3	3	1	21.68413444
2	1	0	2.72694418
3	1	0	181.5224891
3	1	0	67.81220225
3	3	0	15.47458685
2	1	0	41.46269343
3	3	1	14.52179913
1	1	0	35.35171009
1	1	0	31.50770444
2	1	0	80.52699018
1	1	0	23.1954529
2	1	0	95.08164405
3	3	1	2.924072675
2	1	0	15.24460361
2	1	0	71.13053192
2	1	0	18.13582153
2	1	0	30.32493347
2	1	0	103.7552978
2	3	0	91.56618589
2	1	0	11.6962907
2	3	1	65.41380557
2	1	0	143.936656
1	1	0	39.29427999
3	1	0	45.50382758
2	1	1	33.74182738
4	1	1	12.61622368
3	1	0	12.58336893
2	1	0	85.15950981
2	2	0	48.460755
1	1	0	60.2884647
3	1	0	65.18382232
2	1	1	47.17941979
1	1	0	94.26027532
3	1	0	106.219404
4	3	0	0.328547492
2	1	0	76.51871078
3	1	0	7.063771068
2	1	0	73.43036436

3	3	0	83.74675559
2	1	0	22.70263167
2	1	0	109.6691527
3	1	0	12.45194993
3	1	0	77.20866051
1	1	1	68.27216874
3	1	0	72.54328613
2	1	0	76.81440352
2	3	1	3.975424648
2	3	1	63.11397312
2	3	1	52.6004534
3	3	0	55.62309032
2	1	1	88.41212997
2	1	0	76.65012978
3	2	1	60.51844794
3	3	1	10.77635772
2	1	0	29.66783849
1	1	1	14.22610638
3	2	0	123.4352926
3	3	0	72.54328613
1	1	0	111.4761639
1	1	0	66.92512403
1	1	0	85.58662155
2	1	0	20.63278247
2	1	0	121.1354601
2	1	0	37.58583303
1	1	1	67.08939777
2	1	0	93.40605184
3	3	0	0.262837993
2	1	0	75.7959063
1	1	0	15.34316785
2	1	0	119.9526892
3	1	0	59.59851497
2	1	0	81.90688964
3	1	0	36.53448106
2	1	0	103.4267503
3	3	0	5.913854848
2	1	0	93.83316358
1	1	0	105.2337615
2	1	0	116.174393
2	3	1	26.48092782
2	1	0	40.54276046
2	1	0	68.46929724
3	1	0	65.57807931
2	1	0	14.91605612
2	3	1	1.54417321
2	3	0	114.1702533
3	1	0	20.27138023
2	1	0	110.0634097
2	1	0	15.04747511
2	1	0	29.66783849
2	1	0	22.27551993
3	3	1	93.3731971
2	1	0	18.20153103
3	3	1	100.0755659
3	3	0	46.32519631
2	1	0	73.43036436
1	1	0	30.02924073
1	1	0	38.63718501

3	3	0	68.30502349
2	1	0	80.36271643
2	1	0	65.11811282
1	1	1	115.9444098
1	1	0	164.2737458
3	1	0	139.2055722
2	3	1	39.42569898
4	1	1	8.14797779
1	1	0	49.38068798
2	1	0	0.952787725
1	1	0	120.4455104
1	1	0	66.07090055
2	1	0	60.87985018
2	1	0	180.3068634
2	1	0	59.43424122
1	1	0	3.384039163
3	1	0	7.293754312
2	1	0	132.2732201
2	1	0	0.624240234
4	3	1	3.942569898
3	1	0	61.04412393
2	1	0	97.71002398
1	1	0	23.29401715
3	1	0	41.49554818
1	1	0	5.092486119
1	1	0	117.488583
1	1	0	105.2337615
2	1	0	78.03002924
2	1	0	11.66343595
2	1	0	13.07619016
2	1	0	58.58001774
2	1	0	0.887078227
2	1	0	54.24319085
2	1	0	56.2144758
2	1	0	57.66008477
2	1	0	49.97207346
2	1	0	47.21227453
2	1	0	59.40138647
4	3	1	8.969346519
2	3	0	120.5112199
2	1	1	93.3074876
3	1	0	83.74675559
2	1	0	175.6743437
2	1	0	64.0996156
2	3	0	30.9491737
2	1	0	167.8549134
3	1	0	65.9723363
1	1	0	17.54443605
1	3	0	27.30229655
3	1	1	86.17800703
2	1	0	13.5033019
3	3	0	61.83263791
2	1	0	71.16338667
2	1	0	73.75891185
2	1	0	107.2050465
3	3	1	5.946709597
2	3	1	16.98590531
2	1	0	88.60925847
1	3	0	66.1694648

2	1	0	3.515458159
3	1	0	83.81246509
2	1	0	2.201268193
1	1	0	83.97673884
3	2	0	76.05874429
2	3	0	100.5355324
2	1	0	0.952787725
2	1	0	92.42040937
2	1	0	28.55077701
2	1	0	100.3055492
2	1	1	26.41521832
2	1	0	53.22469363
2	1	0	48.78930249
2	1	0	95.87015803
2	1	0	54.50602885
2	3	0	46.16092256
2	1	0	22.66977692
2	1	0	65.51236981
2	1	0	90.41626967
1	1	0	140.1255051
1	1	0	36.53448106
2	1	0	23.3925814
3	1	0	11.79485495
2	1	0	119.0656109
2	1	0	94.42454907
3	1	0	2.72694418
2	3	1	41.39698393
2	1	0	3.48260341
3	1	0	85.32378355
1	1	0	53.09327463
1	1	0	15.90169859
2	1	0	70.73627493
2	1	0	1.347044715
3	3	1	92.74895686
3	1	0	80.42842593
4	1	0	10.61208398
4	1	1	8.837927522
3	3	1	24.14824063
2	2	0	80.00131419
2	1	0	115.517298
2	1	0	15.93455334
3	1	0	59.10569373
2	1	0	128.7577619
2	1	0	153.7930808
1	1	0	15.40887735
2	1	0	88.80638696
2	1	0	43.17114039
1	1	0	88.74067746
3	1	0	9.692151
2	1	0	51.68052042
2	1	0	102.9010744
1	1	0	129.1520189
2	1	0	84.92952656
2	1	0	92.32184512
2	1	0	107.8949962
1	1	0	127.1150245
2	1	0	35.15458159
4	1	0	52.00906791
2	1	0	113.1846108

2	1	0	64.13247035
1	1	0	12.12340244
2	1	0	0.624240234
2	1	0	55.22883333
2	3	0	94.06314683
2	1	0	7.425173309
4	3	0	23.49114565
2	1	0	36.43591681
2	1	0	78.22715774
1	1	0	17.28159805
2	1	0	75.23737556
2	1	0	31.04773795
1	1	0	88.21500148
3	3	1	102.4411079
1	3	0	108.2892532
1	1	0	78.62141473
2	1	0	68.73213523
1	1	0	54.07891711
2	1	0	64.0339061
2	1	0	87.360778
2	3	0	51.84479416
1	1	0	142.3267733
